# Recurrent subacute visual loss presenting in a 52-year-old Caucasian woman with chronic relapsing inflammatory optic neuropathy: a case report

**DOI:** 10.1186/1752-1947-6-15

**Published:** 2012-01-16

**Authors:** Amrit Samra, Jason Ramtahal

**Affiliations:** 1 Department of neurology, South Devon Healthcare NHS Foundation Trust, TQ2 7AA, UK

## Abstract

**Introduction:**

Chronic relapsing inflammatory optic neuropathy is a recently described form of recurrent isolated subacute optic neuropathy. The condition is highly responsive to systemic steroid treatment and prone to relapse on steroid withdrawal. A complete work up for demyelination, autoimmune disease and sarcoidosis must be made before considering chronic relapsing inflammatory optic neuropathy.

**Case presentation:**

We describe the case of a 52-year-old Caucasian woman who presented with isolated subacute optic neuropathy. There was no evidence of demyelination, autoimmunity or sarcoidosis. There was an abrupt and prompt response to systemic corticosteroids and a relapse of the condition on steroid withdrawal.

**Conclusions:**

Chronic relapsing inflammatory optic neuropathy requires careful consideration and differentiation from demyelinating optic neuritis and ischemic optic neuropathy since the treatment is different and the outcome without treatment is likely to be poor. The importance of identifying these patients has considerable clinical implications as the condition is highly responsive to steroids.

## Introduction

Chronic relapsing inflammatory optic neuropathy (CRION) is a recently described form of recurrent isolated optic neuropathy. The condition comprises a characteristic clinical syndrome of subacute optic neuropathy with prominent pain in which there is a clear and prompt response to treatment with systemic steroids but a relapse on steroid withdrawal [1]. The condition does not exhibit any evidence of additional neurological deficits, sarcoidosis or systemic autoimmune diseases. Kidd *et al. *in their report of 15 patients described a clinical syndrome of unilateral or bilateral optic neuropathy characterized by pain followed by subacute visual loss in keeping with an inflammatory cause [1]. Treatment with corticosteroids induced abrupt relief of pain and restoration of visual acuity. Following steroid withdrawal patients tended to relapse, warranting in most cases long term immunosuppression.

## Case presentation

A 52-year-old Caucasian woman with a past medical history of severe hypertension and non-insulin dependent diabetes mellitus presented with bilateral subacute visual loss which progressed over eight to 10 days to perceiving only light in the left eye and only hand movements in the right eye. The visual loss was preceded by dull ocular pain which persisted after the onset of visual loss. The patient experienced pain on eye movements but had no double vision. The patient did not have any temporal artery tenderness and temporal pulses were palpable bilaterally.

On fundoscopic examination, both optic discs were swollen and no retinopathic changes of diabetes or hypertension were evident. There was no evidence of venous engorgement. There was reduced color vision in both eyes and a left relative afferent papillary defect was present. Visual field mapping showed a left central scotoma and a normal right sided visual field. The patient's blood pressure was 152/80 on admission and lower on subsequent readings during her hospital stay. The remaining neurological examination was unremarkable. There was nothing in her history or physical examination suggestive of connective tissue disease or sarcoidosis. Initial blood tests showed a normal full blood count, normal urea and electrolytes (U&Es), normal liver function tests, a normal C-reactive protein (CRP; < 1) and plasma viscosity and a raised glycated haemoglobin (HbA1c) of 7.3% (normal 4 to 6.1). Chest X-ray was normal. A lumbar puncture was performed which yielded cerebrospinal fluid (CSF) with normal white and red blood cells, a normal angiotension converting enzyme (ACE) level, no oligoclonal bands and a marginally elevated protein level of 467.7 mg/L (normal 150 to 450 mg/L). A magnetic resonance imaging (MRI) scan of her brain and spinal cord was normal.

A provisional diagnosis of acute ischaemic optic neuropathy was made by the general medical team and the patient was started on aspirin with omeprazole cover. On review by the neurology team a day later the cause was thought to be inflammatory rather than ischemic. She received a three day course of intravenous methyl prednisolone. Vision improved to 6/6 in both eyes within two days with full restoration of color vision and visual field defects. Ten days later however, vision deteriorated again in the right eye to 6/36 with a temporal peripheral field loss. The relative afferent papillary defect had now switched to the right eye. The patient received 500 mg oral methyl prednisolone for five days. Her visual acuity and visual fields returned to normal within two days.

A subsequent auto-immunity screen including aquaporin-4 antibodies was negative. Visual evoked potentials performed twelve days after the onset of the second episode of visual loss were delayed bilaterally (left > right) indicating bilateral optic nerve dysfunction. Her pattern electroretinograms, brainstem evoked potentials, median nerve and posterior tibial somatosensory evoked potentials were normal bilaterally.

There was no evidence of Leber's hereditary optic neuropathy on genetic molecular analysis. The m.11778G > A, m.3460G > A, m.14484T > C mitochondrial mutations were tested for and not detected in our patient.

A chest computed tomography (CT) was normal and did not show any hilar adenopathy or any other features of sarcoidosis.

Serial MRIs over the next four months were normal without any inflammatory lesions and oligoclonal bands were negative which excluded multiple sclerosis.

## Discussion

Our patient presented with subacute visual loss, the general medical team made a provisional diagnosis of acute ischemic optic neuropathy. When reviewed by the neurology team the pathology was deemed to be optic neuritis, however there was no evidence for any demeyelinating or autoimmune process. Sarcoidosis was also ruled out.

There was an excellent and prompt response to corticosteroids and a dramatic deterioration of vision following steroid withdrawal consistent with CRION. Our case report highlights the importance of the prompt recognition of CRION. The condition is an extremely rare cause of visual loss caused by optic neuropathy and if missed it can have sinister consequences such as permanent visual loss.

## Conclusion

The clinical picture of the patient was similar to those described by Kidd *et al. *in terms of subacute onset of visual deficit, significant pain, excellent response to steroids and visual deterioration following steroid withdrawal [1]. The importance of identifying these patients has considerable clinical implications as the condition is highly responsive to steroids. CRION requires careful consideration and differentiation from demyelinating optic neuritis and ischemic optic neuropathy since the treatment is entirely different and the outcome without treatment is likely to be poor. A complete work up for demyelination, autoimmune disease and sarcoidosis must be made before considering CRION. At present there are no systemic studies evaluating the duration and intensity of immunosuppression required in patients with CRION [2].

## Consent

Written informed consent was obtained from the patient for publication of this case report and any accompanying images. A copy of the written consent is available for review by the Editor-in-Chief of this journal.

## Competing interests

The authors declare that they have no competing interests.

## Authors' contributions

JR examined the patient, analyzed and interpreted investigative data. AS examined the patient, performed a literature review and contributed in writing the manuscript. Both authors read and approved the final manuscript.
